# Delivering the Thinking Healthy Programme as a universal group intervention integrated into routine antenatal care: a randomized-controlled pilot study

**DOI:** 10.1186/s12888-022-04499-6

**Published:** 2023-01-06

**Authors:** Perran Boran, Melike Dönmez, Ezgi Barış, Mahmut Caner Us, Zeynep Meva Altaş, Anum Nisar, Najia Atif, Siham Sikander, Seyhan Hıdıroğlu, Dilşad Save, Atif Rahman

**Affiliations:** 1 Marmara University, School of Medicine, Division of Social Pediatrics, Istanbul, Turkey; 2 Marmara University, School of Medicine, Department of Psychiatry, Istanbul, Turkey; 3 Marmara University, Institute of Health Sciences, Social Pediatrics Doctorate Program, Istanbul, Turkey; 4 Marmara University, School of Medicine, Department of Public Health, Istanbul, Turkey; 5 School of Public Health, Xi’an Jiaotong University, Xi’an, China; 6 Human Development Research Foundation, Islamabad, Pakistan; 7 University of Liverpool, Department of Primary Care and Mental Health, Waterhouse Buildings Block B, Liverpool, L69 3LH UK

**Keywords:** Perinatal depression, Psychological interventions, Thinking healthy program

## Abstract

**Background:**

Women with perinatal depression and their children are at increased risk of poor health outcomes. There is a need to implement non-stigmatizing interventions into existing health systems which reduce psychosocial distress during pregnancy and prevent perinatal depression. We adapted the WHO-endorsed Thinking Healthy Programme (THP) to be delivered universally to all women attending routine online pregnancy schools in Istanbul, Turkey. This study aimed to evaluate the feasibility and acceptability of this intervention.

**Methods:**

This mixed-methods study incorporated a two-arm pilot randomized controlled trial and qualitative evaluation of the feasibility and acceptability of the adapted THP – Brief Group version (THP-BGV) to a range of stakeholders. We recruited pregnant women at 12-30 weeks’ gestation through pregnancy schools within the University Hospital’s catchment area. Women in the intervention arm received five online sessions of the THP-BGV delivered by antenatal nurses. The intervention employed principles of cognitive behaviour therapy to provide psychoeducation, behaviour activation, problem-solving strategies and group support to participants. In the control arm, women received usual care consisting of routine online educational pregnancy classes aided by the antenatal nurses. The women were assessed for depressive symptoms with the Edinburgh Postnatal Depression Scale at baseline and 4-6 weeks post-intervention and also evaluated for anxiety, perceived social support, partner relationship, level of disability and sleep quality. In-depth interviews were conducted with women and other key stakeholders.

**Results:**

Of the 99 consecutive women referred to the pregnancy schools, 91 (91.9%) were eligible and 88 (88.8%) consented to participate in the study and were randomized. Eighty-two (83%) completed the final assessments. Our main findings were that this preventive group intervention was feasible to be integrated into routine antenatal educational classes and it was valued by the women and delivery-agents. While the study was not powered to detect differences between intervention and control conditions, we found small trends towards reduction in anxiety and depressive symptoms favoring the intervention arm. No serious adverse events were reported.

**Conclusions:**

Given the paucity of preventive interventions for perinatal depression in low and middle-income countries, a fully powered definitive randomized controlled trial of this feasible and acceptable intervention should be conducted.

**Trial registration:**

The study was registered at Clinical Trails.gov (NCT04819711) (Registration Date: 29/03/2021).

**Supplementary Information:**

The online version contains supplementary material available at 10.1186/s12888-022-04499-6.

## Background

Globally, perinatal depression (PND) is the most common contributor to pregnancy-related morbidity and mortality, with an onset occurring in 33.4% women during pregnancy and up to 40% within the first postnatal year [1, 2]. Turkey with a population of approximately 84.6 million is one of the largest countries in Europe and Central Asia region [3]. Studies from the country show that 19.1% of new mothers had depressive symptoms in the early postpartum period [4]. PND bears negative physiological and psychological outcomes for both mother and child. It can adversely impact the critical period of fetus’ early brain development [5–7] and is associated with poor cognitive and emotional development and behavioral problems in later life [1, 8, 9]. Yet, women who are depressed are less likely to seek help and the majority do not receive treatment [10, 11]. Turkey has attained significant improvements in provision of mental health care services, through their Mental Health Action Plan, initiated in 2006 and later updated for 2020-2023 [12]. Community based mental health services model was adopted in 2011 and by 2020 there were 177 Community Mental Health Centers (CMHCs), and Healthy Lifestyle Centers (HLCs) in 78 provinces of Turkey [12]. Despite these efforts, workforce in mental health is still below the desired levels compared with EU countries [12].

Considering the high prevalence of PND, its adverse impact, and lack of mental health workforce to address this issue, there is an urgent need to develop and implement non-specialist delivered preventative interventions for the condition. Cognitive Behavioral Therapy (CBT), provided in different delivery formats, is an evidence-based intervention recommended as first line psychological intervention for both treatment and prevention of PND [13–15]. CBT based preventative interventions enhance coping strategies to avoid depression symptoms becoming severe [16]. Furthermore, such interventions when delivered universally in group settings help overcome fear of stigma – a significant barrier in accessing mental health services [17, 18]. A key strategy for scale-up of evidence-based interventions is to adapt and integrate them into existing platforms [19]. The Thinking Health Programme (THP) is an example of a CBT-based psychosocial intervention that was designed to be delivered by community health workers in Pakistan without previous training in mental health [20]. It is the first psychosocial intervention to be incorporated into the World Health Organization’s flagship Mental Health Gap Action Programme (mhGAP), after randomized controlled trials demonstrated a strong effect size of the intervention [21].

Turkey prioritizes the provision of effective, high quality health services in terms of maternal and infant health, and provides ‘antenatal pregnancy schools’ where women are invited to attend 5 weekly group sessions that incorporate education about pregnancy and newborn care. During the COVID19 pandemic, these classes were adapted into an on-line platform where women could access training videos on the website of the General Directorate of Public Hospitals of the Ministry of Health (MoH) [22, 23]. Integrating THP into this existing service for perinatal health has the potential to fill the important gap of mental health care in Turkey. In view of this, we have developed an on-line brief group version of the THP (THP-BGV) designed to be integrated into the routine on-line antenatal pregnancy classes as a preventative intervention [24]. The Medical Research Council (MRC) guidelines suggest pilot studies resembling the intended trial having a control group and randomization on a smaller scale to be used prior to any large-scale definitive trial designed to evaluate the effect of complex interventions [25]. The aim of this study was to pilot this adapted group intervention to evaluate its feasibility before a future definitive randomised controlled trial (RCT) and wider scale implementation.

## Methods

### Study design

This mixed-methods study incorporated a two-arm pilot randomised controlled feasibility trial, and qualitative evaluation of the acceptability of the THP-BGV to a range of stakeholders. We tested the overall design, number of participants eligible, recruitment rates, willingness of the women and the facilitators to participate in the study, time taken to complete measurements and their acceptability, randomization process, data collection procedures, intervention acceptability, session adherence rates, and follow-up (response rates, adherence, dropout rates).

### Settings and participants

The study was conducted in Istanbul, which constitutes approximately 18.5% of Turkey’s population [3], in socioeconomically deprived urban populations from the catchment area of the Marmara University Pendik Training and Research Hospital. This population is characterized by low literacy (about 30% women not attended school), high rates of internal migration, low socioeconomic status and poor maternal and child health indicators. Marmara University Hospital, with the support of Turkish MoH, operates ‘antenatal pregnancy schools’ for low-income women living in these municipalities. The routine groups usually consist of 8-10 women. Our brief group intervention was incorporated into these routine antenatal care systems. The study took part during the COVID-19 pandemic when hospitals as well as public transport systems were facing lock-downs. As the study was conducted during the COVID pandemic, routine antenatal pregnancy schools were run online and arrangements were made to deliver the THP-BGV classes online incorporated into routine classes. Before online adaptation, focus group discussions were performed to explore women’s views and needs about online classes [24]. Main adaptations to account for online delivery included user experience and accessibility such as providing data credits to participants and instructions on how to attend the sessions. Other adaptations were to decrease the number of original sessions to comply with the number of routine antenatal classes, preparation of PowerPoint slides with culturally adapted illustrations to present in online sessions, supervisions by THP trainers who are not necessarily mental health specialists to be able to sustain the model.

The sessions were delivered via Zoom and the meeting link was only shared with the participants of the related sessions to ensure confidentiality. All personal data was regarded as being strictly confidential and participants' phone number was used only for the purposes of sending reminders before the sessions by the site coordinator and later for the assessments by the research assistants.

The study was conducted following the principles in the Declaration of Helsinki on experimentation involving human subjects; the study protocol was approved by Marmara University ethics review board (09.2018.389) and Liverpool (8722), and registered at Clinical Trails.gov (NCT04819711-Registration Date: 29/03/2021).

The study participants included all consecutive pregnant women who had been enrolled to attend these online pregnancy school classes between April 8th 2021 to September 8th 2021.

### Recruitment procedure and inclusion/exclusion criteria

Women enrolled in the pregnancy school programme were provided basic information about the study by the pregnancy school nurse and asked for permission to be contacted by a research assistant (RA). Upon receiving permission, their contact details were shared with the RA to assess eligibility. Inclusion criteria were: pregnant women aged 18 years or over; between 12 and 30 weeks of gestation, and; intending to attend all 5 on-line sessions of the pregnancy school programme. Exclusion criteria included women who were currently receiving any form of mental health care or reported suicidal ideation. Women who fulfilled the eligibility criteria were given information about the trial and all participants providing online informed consent were included in the trial.

### Randomization

The generation of random allocation sequence was done by an independent statistician at the University of Liverpool, who was not involved in the enrolment of participants in the trial. Simple numbers were provided to the local Principal Investigator (PI) through mail who assigned the participants to THP-BGV or Treatment as Usual. Randomization was performed after the baseline assessments were completed. The site coordinator was informed of the allocation status, and contacted both the participants and the designated nurse to inform them of the randomization outcome. The pregnancy school nurses and the participants were requested not to discuss their treatment allocation with the assessment team while completing the post-intervention and follow-up assessments. Other than outcome assessors, it was not possible to blind antenatal nurses in participating centers. Blinding for the participants was not possible because they can easily predict the allocated assignment due to the longer duration of the intervention sessions.

### Intervention

The intervention is based on the Thinking Healthy Programme [20, 21, 26], an evidence-based intervention that employs cognitive behaviour therapy strategies to achieve three main goals: a) To identify and modify maladaptive styles of thinking and behaving – in particular those leading to poor self-esteem, inability to care for their infants, and disengagement from social networks; b) behavioural activation – adopting behaviours, such as self-care, attention to diet, and positive interactions with the infant, and practicing these between sessions; c) problem-solving to overcome barriers to practicing such strategies. The adapted THP-BGV employs similar strategies adapted for prevention rather than treatment of perinatal depression [24]. An additional element is support from the facilitator and other group members. The intervention consists of five group sessions: 1) Engagement and introduction to the programme; 2) psychoeducation and problem-management skills; 3) focusing on one’s personal health and well-being; 4) establishing the mother-infant bond, and; 5) reactivating relationships with others and closing the therapy. The intervention group sessions lasted one hour, on average, except the introduction session which lasted 90 minutes. The intervention is designed to be delivered on-line or face-to-face by facilitators who receive a brief four to five-day training, strengthened by experiential learning and monthly half-day facilitated group supervision. The programme is fully manualized, and includes instructions for the delivery of each session with culturally appropriate pictorial illustrations aimed at reinforcing key messages and encouraging family involvement.

THP-BGV was designed to be integrated into routine antenatal education classes delivered by nurses. The nurses have no formal mental health care training. A training manual was developed with the aim of standardizing the training and delivery procedures [24]. The Bernal Framework for adaptation was used to adapt THP for universal use in the group settings [27]. The original THP manual was previously translated into Turkish by PB and adapted for the five sessions of antenatal pregnancy school [24]. Original pictures were replaced with culturally appropriate illustrations. In total, 15 hours of training (spread over 4 days) were conducted via zoom by the THP trainers (PB and MD). The trainings aimed to: (a) educate the nurses on psychosocial factors impacting mother and child health during the perinatal period, (b) learn and practice basic counselling skills and (c) understand the intervention principles, contents and its delivery mechanisms. Different training methods were used, including lectures, discussions and activities, use of case scenarios, sharing personal experiences and role-plays. The role-plays aimed at practicing counselling skills and dealing with challenging situations. The intervention material consisting of the session power-point presentations, and job-aids were given to the nurses to assist them in the delivery of the intervention.

The intervention group received both the routine antenatal sessions and an additional one-hour THP session weekly.

The site coordinator informed the participants of their allocation status, and provided information about the on-line delivery platform. A link for each session was shared with the participants by the site coordinator. A WhatsApp group was set up by the designated pregnancy school nurse to remind participants of the date and time of the sessions.

Full details of the THP-BGV are available in the adaptation paper [24].

### Intervention Fidelity

Intervention fidelity was ensured through weekly supervision of the antenatal nurses by trained THP supervisors (PB and MD). The nurses were provided group supervision lasting up to 2 hours and entailed reviewing the progress of intervention delivery, and additional refresher training on intervention components. The THP supervisors in turn received monthly supervision by a Master Trainer (NA) online for approximately 1 hour. In addition, antenatal nurses had day-to-day support of their supervisor at the hospital. Intervention fidelity was tested through independent observations of sessions by the THP supervisors using the Enhancing Assessment of Common Therapeutic factors tool (ENACT) [28]. ENACT uses a three-point Likert scale to score an observed interaction on 18 dimensions, with a total score of 54, and a higher score indicating greater competence. For the present study, an adapted ENACT composed of 17 items was used (excluding item 11 as this was related to appropriate involvement of family members).

### Treatment as usual

Participants randomized to the control arm attended the 5 sessions of the routine online educational group pregnancy classes facilitated by a designated nurse with no formal training in psychological interventions. The routine pregnancy classes during COVID-19 provided on-line access to education about pregnancy, birth, new-born care and included basic information about PND, and how to identify it and seek help. The women in the control arm were able to access all usual care and support offered by the participating hospitals.

### Primary and secondary outcome measures

Our primary outcome was the level of depressive symptoms measured with the Edinburgh Postnatal Depression Scale (EPDS) [29–31] at baseline and 4-6 weeks post-intervention.

Secondary outcomes after 4-6 weeks post-intervention included the Patient Health Questionnaire-9 (PHQ-9) [32, 33], General Anxiety Disorder-7 (GAD-7) [34, 35], Brief-Coping Orientation to Problems Experienced (COPE) [36, 37], Multidimensional Scale of Perceived Social Support (MSPSS) [38, 39], Relationship Assessment Scale (RAS) [40–42], Pittsburgh Sleep Quality Index (PSQI) [43, 44], and the World Health Organization Disability Assessment Schedule (WHODAS) [45–47].

Assessment forms were completed in 30 to 45 minutes. All the instruments used have been validated to Turkish and found reliable. Detailed description of the assessment instruments has been provided in the Table 1 and as supplementary material.

**Table 1:** Description of the assessment instruments

	Point of application	Number of items	Scoring range	Scoring	Explanation
**EPDS** [29–31]	At baseline and 4-6 weeks post-intervention	10	0-30	> 12 considered as cut-off	Developed for detection of symptoms of psychosocial distress during pregnancy and in the postnatal period
**GAD-7** [34, 35]	At baseline and 4-6 weeks post-intervention	7	0-21	> 8 considered as cut-off, scores of 0-4, 5-9, 10-14 and 15-21 represents minimal, mild, moderate and severe anxiety respectively	Instrument that measures generalized anxiety disorder
**PHQ-9** [32, 33]	At baseline and 4-6 weeks post-intervention	10	0-27	A score of 10 or above indicates major depression, and scores of 1-4, 5-9, 10-14, 15-19 and 20-27 represents minimal, mild, moderate, moderately severe, and severe depression respectively	The instrument can help to diagnose depression as well as measure th severity. Both the PHQ-9 and EPDS are reliable and valid scales for assessment of depression during perinatal period whereas PHQ-9 captures somatic symptoms, while EPDS detects depressive symptoms comorbid with anxiety
**Brief-COPE** [36, 37]	At baseline and 4-6 weeks post-intervention	28	28-112	No cut-off point for total and subscales, higher scores indicate increased utilization of the specific coping strategy	The scales of the Brief-COPE help to asses’ behaviors of people against stress, included suppression of competing activities, planning, positive reframing, acceptance, humor, religion, using emotional support, using instrumental support, self-distraction (mental disengagement), denial, venting, substance use, behavioral disengagement, restraint coping
**PSQI** [43, 44]	At baseline and 4-6 weeks post-intervention	9	0-21	The global score > 5 indicates poor sleep quality and higher scores indicate worse sleep quality	Combined to form seven component scores including; subjective sleep quality, sleep latency, sleep duration, habitual sleep efficiency, sleep disturbances, use of sleeping medication, and daytime dysfunction. The seven component scores are then added to yield one global score
**RAS** [40–42]	At baseline and 4-6 weeks post-intervention	7	7-35	Scoring is kept continuous. Higher scores indicate higher levels of relationship satisfaction	Used to assess subjective satisfaction with a given relationship
**WHODAS** [45–47]	At baseline and 4-6 weeks post-intervention	12	0-100	Range between 0 and 100 where 0 = no disability 100 = full disability, higher scores indicate higher disability of loss of function	It was used to measure disability and functional impairment. The questionnaire covers six domains of functioning: cognition (understanding and communication), mobility, self-care, getting along with others, life activities (work and household roles), and participation in society
**MSPSS** [38, 39]	At baseline and 4-6 weeks post-intervention	12	12-84	No cut-off point for total and subscales, higher scores indicate higher levels of perceived social support	This scale was developed to measure perceived social support from family, friends, and significant other

Abb. *EPDS* Edinburgh Postnatal Depression Scale, *GAD-7* General Anxiety Disorder-7, *PHQ-9* Patient Health Questionnaire-9, *COPE* Brief-Coping Orientation to Problems Experienced, *PSQI* Pittsburgh Sleep Quality Index, *RAS* Relationship Assessment Scale, *WHODAS* World Health Organization Disability Assessment Schedule, *MSPSS* Multidimensional Scale of Perceived Social Support

### Data collection

Assessments were conducted over the phone. Data were collected at two timepoints: at baseline and 4-6 weeks post-intervention, by RAs blind to treatment allocation status of the study participants. The questionnaires were administered using a smartphone and responses were recorded by the RAs onto an online data capture form. This online form was access protected and all data imputed by the RAs were encrypted through a two-step authentication process.

Within 24 hours, the site coordinator reviewed all assessments from the previous day to check for completeness and accuracy. The assessment forms contained only the case ID number of the participant to ensure confidentiality and anonymity of the data. Data were downloaded into a database at Marmara University, backed-up, and secured in a hard drive. At the end of the trial, data were collated into a dataset using the SPSS statistical package for analyses.

The site coordinator referred women who were screened positive for depressive symptoms (EPDS scores > 12) to an on-call psychiatrist for clinical assessment and further management as per hospital routine practice.

### Process evaluation

Qualitative data sources included field notes taken by local site coordinator and in-depth interviews conducted with the participating women and nurses. In-depth interviews were carried out after the end-point outcome assessments. Women’s motivation for participation, their experience of group work, views on the intervention (timing, duration, frequency and content), facilitators’ experience of delivering the group sessions, perceived benefits of the programme, and how the intervention could be improved were explored in the interviews.

Participants for qualitative interviews were purposively selected by the site coordinator through the study database to ensure heterogeneity in reference to number of sessions attended. All selected participants agreed to be interviewed except women who attended no sessions following recruitment. Informed consent was obtained online. To avoid potential bias, two independent researchers (SH, DS) who were not involved in the study conducted the in-depth interviews online with twelve women and two nurse facilitators after reaffirming their consent verbally. The interviews were aided by a pre-defined semi-structured interview guide based on previous research on cultural adaptation studies of the original THP [48]. An interview guide was developed to explore the accessibility, acceptability, feasibility and implementations of the THP-BGV. It was also informed by the THP adaptation and feasibility study conducted in Pakistan and India [48].

The interview guide was pilot tested before being finalized to ensure that questions within each dimension were generating relevant information. All interviews were audio recorded, transcribed and checked back against the original audio recordings for accuracy. Interviews lasted between 45 to 60 minutes.

### Sample size and data analyses

Our proposed sample size of 60 (30 participants in each arm) was based on the pragmatics of recruitment and the necessities for examining feasibility, which would allow us to assess our recruitment procedures and attrition rates, and evaluate the feasibility of integrating the intervention into routine antenatal classes.

All quantitative analyses were conducted using IBM SPSS Statistics software (version 28.0, IBM Inc., United States). Feasibility data such as recruitment rates, adherence to the intervention were summarized and presented as percentages. Chi-square test was applied to identify differences in the distribution of categorical variables by groups, and Mann-Whitney U test for continuous variables. An overall 5% type-I error level was used to infer statistical significance. Binary logistic regression was used to assess the associations between session attendance and sociodemographic characteristics of the participants. Adjusted odds ratios (ORs) and 95% confidence intervals (CIs) were reported. The Wilcoxon test was performed to test the significance of pairwise differences between pre- and post-intervention scores.

Qualitative analysis was done using a thematic framework approach [49]. This approach was used because it is a systematic procedure to illustrate themes in large datasets. Data analysis utilized all the five steps of the framework analysis method: familiarization with data, initial coding, searching for themes, reviewing and defining themes, and interpretation of the data [49]. Transcripts were read and re-read to familiarize with the data by two independent researchers. Codes were generated and highlighted to identify patterns, and then collated into potential themes. The themes were reviewed and any disagreement was discussed with the research team to clarify the findings. The process of analysis was supervised by the local PI. The final report with the relevant quotes were translated into English. The report was supported by the field notes from the research team.

## Results

**Fig. 1:**
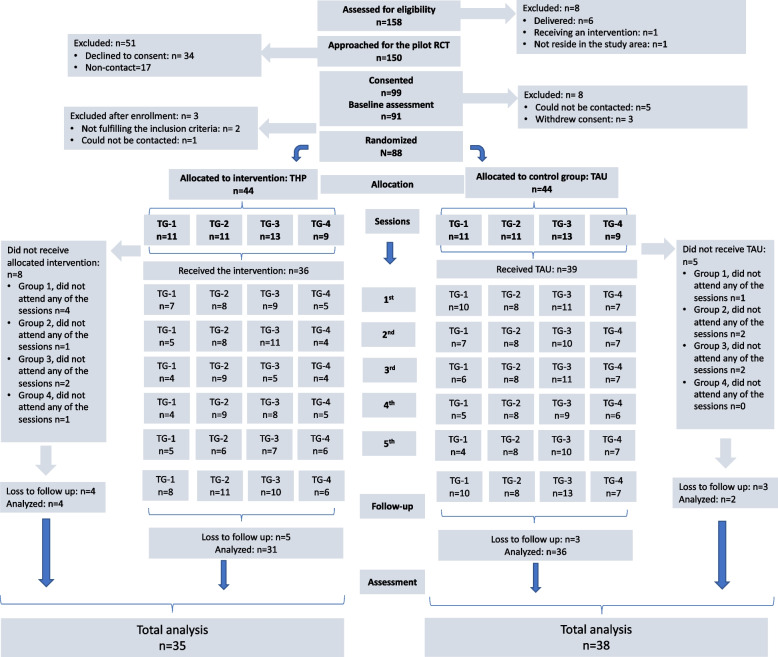
Participant Flow Diagram. Abbreviations: *THP* Thinking Healthy Programme, *TAU* Treatment as Usual

Out of 158 eligible women, 8 did not meet the study inclusion criteria, and 34 (22.6%) declined to participate. The age of the women who refused to participate (26.5 ± 5.0 vs 28.6 ± 4.8 years) and gestational weeks (19.7 ± 5.9 vs 21.1 ± 5.4) were not different from the study participants. Reasons for refusal included: time constraint (*n* = 18); lack of interest (*n* = 4); illness (*n* = 2); partner not wanting participation (*n* = 7), and; problems accessing the internet (*n* = 3). Of the 99 women referred, 91 (91.9%) completed baseline screening and 88 of these were recruited and randomized. Out of 88 women enrolled, 73 women (83%) completed final assessments. The participant flow chart is presented in Fig. 1.

Overall, 76% of the women were enrolled from antenatal clinics, and 24% from social media. Just over 50% had high school or lower education, and 43% were housewives. Internet package were provided for 26% of the women on request. There were no significant differences between the two arms in participant characteristics (Table 2).

**Table 2:** Participant characteristics

	THP (*n*: 44) Mean ± SD	TAU (*n*: 44) Mean ± SD	***p*** value
**Gestational weeks**	20.70 ± 5.54	21.41 ± 5.45	0.549
**Family size**	2.66 ± 1.08	2.73 ± 1.19	0.779
**Parity** (*n*, %)			
Nulliparity	28 (63.6)	31 (70.5)	0.496
Multiparity	16 (36.4)	13 (29.5)	
**Family type** (*n*, %)			
Nuclear	41 (93.2)	41 (93.2)	1.000
Extended	3 (6.8)	3 (6.8)	
**Maternal age**	27.64 ± 4.79	29.61 ± 4.77	0.056
**Paternal age**	31.02 ± 4.48	31.95 ± 4.65	0.341
**Maternal education** (*n*, %)			
No formal education	3 (6.8)	0 (0)	0.282
Primary school	9 (20.5)	10 (22.7)	
High school	13 (29.5)	10 (22.7)	
College/University	19 (43.2)	22 (50.0)	
Graduate (MD, PhD, MS)	0 (0)	2 (4.5)	
**Paternal education** (*n*, %)			
No formal education	2 (4.5)	0 (0)	0.773
Primary school	10 (22.7)	9 (20.5)	
High school	12 (27.3)	12 (27.3)	
College/University	18 (40.9)	21 (47.7)	
Graduate (MD, PhD, MS)	2 (4.5)	2 (4.5)	
**Working status** (*n*, %)			
Not working	32 (72.7)	28 (63.6)	0.360
Working	12 (27.3)	16 (36.4)	
**Internet package** (*n*, %)			
No need	31 (70.5)	34 (77.3)	0.467
Internet supplied	13 (29.5)	10 (22.7)	
**Enrollment** (*n*, %)			
Outpatient clinic	35 (79.5)	32 (72.7)	0.453
Social media	9 (20.5)	12 (27.3)	
**Final assessment (*n*, %)**			
Completed	35 (79.5)	38 (86.4)	0.572
Not completed	9 (20.5)	6 (13.6)	
**Number of attended sessions** (*n*, %)			5
0	8 (18.2)	5 (11.4)	0.134
1	4 (9.1)	4 (9.1)	
2	4 (9.1)	2 (4.5)	
3	5 (11.4)	5 (11.4)	
4	13 (29.5)	6 (13.6)	
5	10 (22.7)	22 (50.0)	

Mean, standard deviation are given for continuous variables, and percent and sample size (*n*) for categorical variables

There was a substantial but statistically non-significant difference in attrition before the start of the intervention, 8 (18.2%) of randomized intervention participants never attended the intervention, versus 5 (11.4%) of control participants (*p* = 0.549). Details of session attendance is given in Fig. 1 and Table 2. In the intervention arm, 36.4% (*n* = 32) women attended all sessions, and 14.8% (*n* = 13) attended no sessions. Session attendance was not different between intervention and control arms. We examined women’s baseline sociodemographic characteristics as predictors of their attendance in the group sessions. In the univariate analyses, higher maternal education and enrolment from social media were significantly associated with higher session attendance, but significance disappeared in regression analyses (Table 3). Parity or depression at baseline was not significantly associated with session attendance.

**Table 3:** Predictor variables for session attendance

Predictors for session attendance	Session attendance low ***n*** (%)	Session attendance high ***n*** (%)	Univariate OR, CI [95%]	Odds Ratio Multivariate logistic regression OR, CI [95%]
*Maternal education*				
University or higher	13 (35.1)	30 (58.8)	0.37 [0.15-0.91]^*^	0.67 [0.24-1.81]
High school or lower	24 (64.9)	21 (41.2)		
*Enrollment venue*				
Outpatient clinic	33 (89.2)	34 (66.7)	4.12 [1.25-13.55]^*^	2.86 [0.81-10.1]
Social media	4 (10.8)	17 (33.3)		
*Parity*				
Nulliparity	22 (59.5)	37 (72.5)	0.55 [0.22-1.36]	
Multiparity	15 (40.5)	14 (27.5)		
Depressed	1 (2.7)	2 (3.9)	1.46 [0.12-	
Not-depressed	36 (97.3)	49 (96.1)	16.83]	
Family size	3.1 ± 1.4	2.4 ± 0.8		0.68 [0.44-1.07]

Abbreviations: *OR* Odds Ratio, *CI* Confidence Interval, ^*^ *p* < 0.005

Factors that were statistically significant in bivariate analysis were then entered in regression model

Hosmer and Lemeshow goodness of fit statistics were used to assess model fit (*p* = 0.471). Nagelkerke R Square = 0.164

**Fig. 2:**
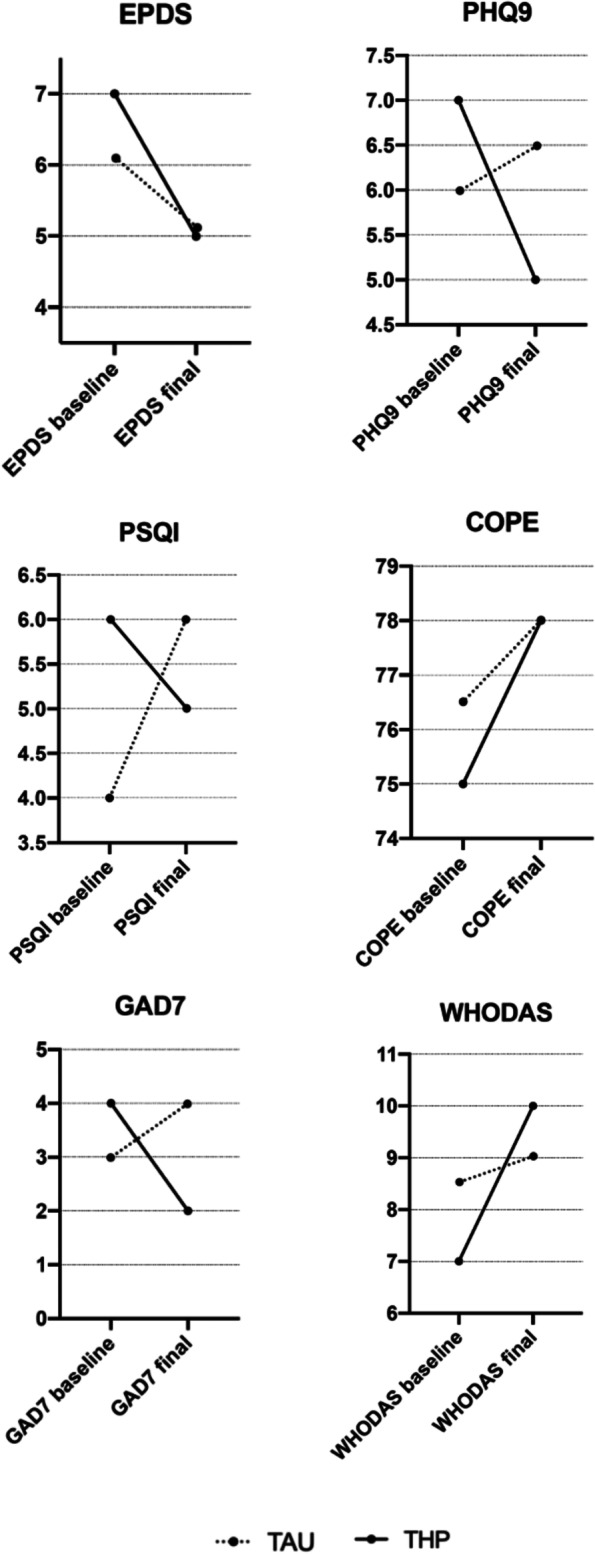
Outcome measures pre to postintervention. Abbreviations: *THP* Thinking Healthy Programme, *TAU* Treatment as Usual

Depressive symptoms were positive in 3.4% of the women as determined by an EPDS score > 12 at baseline. There was a reduction in mean EPDS, GAD-7, PHQ-9 scores and an increase in COPE scores from pre- to post-intervention for the intervention group, while mean scores for the control group did not change from pre to post intervention except WHODAS scores where higher post-intervention scores indicated increased disability or loss of function (Table 4***,*** Fig. 2).

**Table 4:** Outcome measures pre- to post-intervention

	THP (*n*: 35) Mean ± SD	TAU (*n*: 38) Mean ± SD	*p value
**EPDS**			
Baseline	6.17 ± 3.06	5.97 ± 3.39	0.597
1 month post-intervention	4.86 ± 2.90	5.03 ± 4.56	0.393
†*p* value, median difference	***0.025***	*0.063*	
**GAD7**			
Baseline	4.69 ± 3.42	4.26 ± 3.94	0.893
1 month post-intervention	3.23 ± 2.70	4.34 ± 3.51	0.154
†*p* value, median difference	***0.020***	*0.866*	
**PHQ9**			
Baseline	6.94 ± 3.70	6.61 ± 4.10	0.207
1 month post-intervention	5.23 ± 3.95	6.21 ± 3.94	0.190
†*p* value, median difference	***0.011***	*0.474*	
**COPE**			
Baseline	74.51 ± 5.59	77.71 ± 7.25	0.117
1 month post-intervention	77.09 ± 7.26	76.29 ± 9.15	0.925
†*p* value, median difference	***0.039***	*0.494*	
**PSQI**			
Baseline	5.91 ± 2.59	5.82 ± 3.47	0.267
1 month post-intervention	5.66 ± 2.70	5.92 ± 3.47	0.956
†*p* value, median difference	*0.561*	*0.781*	
**RAS**			
Baseline	32.29 ± 3.39	32.53 ± 3.65	0.877
1 month post-intervention	32.71 ± 3.63	32.66 ± 3.15	0.441
†*p* value, median difference	*0.201*	*0.757*	
**WHODAS**			
Baseline	9.51 ± 5.61	8.53 ± 6.45	0.109
1 month post-intervention	10.34 ± 6.66	10.61 ± 6.67	0.868
†*p* value, median difference	*0.315*	***0.043***	

*p value for differences between usual care and intervention groups at baseline and final assessments (Mann-Whitney U test)

† *p* value for the difference in median: baseline/1 months post-intervention in the usual care and intervention groups (related-samples Wilcoxon signed rank test)

*p* value is given in italics

There were no significant group differences in EPDS, GAD-7, PHQ-9, COPE, PSQI, RAS, WHODAS scores between intervention and control group, although there was a trend in the intervention group towards lower depressive symptoms. Depression rates at baseline and final assessments were not different as measured by EPDS and PHQ-9 (Table 4***,*** Fig. 2).

### Qualitative results

A total of 14 in-depth interviews were conducted with 12 women (10 from intervention arm and 2 from control arm) and 2 facilitators. Women interviewed from the intervention arm had received at least 3 sessions. The analysis of the data generated information pertaining to the accessibility, acceptability, feasibility and implementations of the THP-BGV.

The main reasons given for taking part in the study included the need for receiving well-being information during pregnancy (I.a, I.b.); having an opportunity to interact with other women in similar circumstances (I.c), and; ease of access through an online delivery format (I.e). Online delivery of the intervention was found convenient and affordable for working women, women who had difficulty accessing the hospital, and those who were housebound due to child care responsibilities (I.d). Examples of quotes are given in Table 5.

**Table 5:** Qualitative findings and example quotes

Dimension	Themes	Example quotes
I.Accessibility	Factors for participation. (Willingness to become informed parents, socialization, interactive virtual training)	*a. “Awareness is important, I aimed to raise my awareness and become a better parent so my child become a conscious individual.” ÖA, 29 y, housewife, THP*
		*b. “I had no idea about pregnancy, Therefore I was reading books and I was searching for antenatal classes in the hospitals, nearby” GK, 31 y, housewife, THP*
		*c. “I thought that interactive nature of the classes will be good. It is good to have the opportunity to ask questions” CL, 33y, chemical engineer*
		*d. “I have been looking for a childbirth education program but I was worried about transportation as I have a little one. When I heard that it’s online, I immediately wanted to join.” SK, 24y, housewife, THP*
		*e. “We could reach out the working mothers with the online sessions” Nurse Ö*
II.Acceptability	Perceived benefits. (Normalization of feelings and thoughts, improved relationship with significant others, increased maternal self- efficacy and confidence, promotion of healthy lifestyle habits, increased psychosocial well-being, bonding with the baby, dissemination of information, social networking)	*a. “I noticed how bad I am sleeping and that my mental status was not good when I was answering the questions (during assessments)*.” *DT, 22y, housewife, THP*
		*b. “I had negative thoughts since my husband is working in another city and not coming often. I learned how to transform my negative thoughts into positive thoughts. I noticed that my negative thoughts can also impact the health of the baby” 22y, technic service personnel, THP*
		*c. “We were trying to change our unhealthy thoughts with healthy thoughts. Doing this together, we were able to see that other women also were experiencing similar issues.” 28y, housewife, THP*
		*d. “I was so emotional before the sessions. I had negative thoughts which I have learned to change into positive thoughts. I even think that the sessions changed my personality” 25 y, housewife, THP*
		*e. “One of the best things was seeing that other women were also distressed like me. I was feeling guilty like is pregnancy was too much for me but I see that other women were feeling the same” 30 y, municipality worker, THP*
		*f. “I had an abortus after which I was so depressed. I was distressed that I will lose this baby too. I overcome such negative thoughts with the help of this program” 24y, housewife, THP*
		*g. “I had so many question marks in my head. I wonder if I can be enough as a mother? How can I deal with this? When I was told about such a project, I said I was in the right place. I definitely have to do this. …….. There’s been a lot of things that this program added to me” 31 y, worker, THP*
		*h. “I started to talk to the baby and listen music with the baby” 33y, chemical engineer, THP*
		*i. “I took notes and shared them with my pregnant friends. They were very happy with that” 31 y, housewife, THP*
		*j. “The sessions obviously improved my marital relationship. I used to be an easily offended person. Now my husband says that he is surprised that I’m not crying easily anymore. I’m also feeling different than past. I feel emotionally stronger” 22y, housewife*
		*k. “I did breathe exercises outside in clean air and noticed that I can sleep better.” 31 y, worker*
		*l. “I was so stressed and nervous, that I was reflecting these to my husband and friends. Now when I get angry about something I just take a moment and do my breathing exercises and relax” 24y, housewife, THP*
		*m. “I did not know that the breathing exercises can relax my baby, too. When I start doing my breathing exercises my baby begins to move” 29 y, housewife, THP*
		*n. “Watching videos together with the nurse was better. After watching the videos, we were discussing and asking our questions. This was helping with focusing and seeing other points” 24y, housewife, THP*
		*o. “Visualization of the feelings by the charts was useful. Normally you can’t remember if asked how you felt in the last week but in the charts, you can see how your feelings changed” 33y, chemical engineer, THP*
		*p. “It wasn’t just like a training but it was as if the girls have met.” 31y, worker, THP*
		*q. “In general, I was very happy that such a project is being conducted in my country. Most of the publications I read were from other countries and I was thinking why such projects were not done in my country and why we would apply results of other studies in our own country without knowing the results it in our country” 33y, chemical engineer*
III. Feasibility	Appreciation of new knowledge	*a. “Thinking healthy program should be integrated into all pregnancy schools, mothers should be aware that if they feel well, their baby feel well, too.” Nurse*
		*b. “I will use what I have learned in this program in my routine pregnancy classes.” Nurse*
		*c. “As a team, I believe that it is a good work. Calling us, giving information before the training, conducting a survey, I thought that it was a nice and good team at every stage. I had no distrust towards anyone.” 28y, housewife, THP*
IV. Implementation	Proper implementation (content, views of nurses, potential barriers, skills of facilitators)	*a. “The content was rich especially about stress management, mental changes during pregnancy, but there could be more about birth and newborn care” 33y, chemical engineer, THP*
		*b. “The trainer had a good energy that I really appreciated. Even some participants were not active, she kept her energy high” CL, 33y, chemical engineer, THP*
		*c. “She was self-confident. She invited everyone to the conversations.” FÇ, 22 y, technic service personnel, THP*
		*d. “I preferred to leave my camera on. Some participants were doing housework like ironing while the camera is off, but you know you are being listened while the cameras are on. Leaving the cameras off may also demotivate the trainers.” CL, 33y, chemical engineer, THP*
		*e. “I would like to meet the people face to face since a connection was built among us” 31 y, housewife, THP*
		*f. “I think it is better to participate with the partners. Fathers should get involved in raising the child as much as the mothers. In fact, they need more training that us, the mothers. We might not notice the psychological changes that the fathers are experiencing” 33y, chemical engineer, THP*
		*g. “Compared to the face-to-face trainings, in online sessions, focusing is more difficult. Sometimes, somebody lost connection and when you wait her to be connected again you can be distracted.” 33y, chemical engineer, THP*

The in-depth interviews revealed an overall satisfaction with the intervention (Table 5). Most participants found the group intervention very beneficial as it provided them with the opportunity to share and learn from each other’s experiences (II.c, II.e). Most women found the sessions helpful in improving their overall wellbeing through increasing their self-confidence and self-efficacy, while reducing their anxiety and stress about pregnancy and child care (II.f, II.g, II.l). They reported being able to translate their knowledge into practice such as the ability to manage their unhelpful thoughts, which in turn helped with their partner relationship (II.b, II.j). Breathing exercises and mood charts were found particularly helpful (II.k, II.m., II.o.). By monitoring their mood, women learned to understand the relation between their feelings, thoughts and behavior and felt motivated to engage in healthy activities. Another identified benefit of the intervention was the dissemination of information, not only to those who were participating in the intervention but also to the wider circle such as family and friends (II.i). No negative effects of the intervention were noted by the participants or researchers. Tools used during assessments were appreciated since they helped women to notice their mental status and lifestyle habits, while answering questions (II.a).

Facilitators appreciated learning new knowledge and enhancing their skills relevant to their practice, and felt the programme fulfilled a gap in their service (III.a., III.b., III.c.).

Qualitative interviews revealed that participants characterized the facilitators as enthusiastic and encouraging, which was an important indicator of acceptability at the staff level (IVb, IVc). In addition to qualitative findings, intervention fidelity and competency of the facilitators were assessed through direct observation of the sessions and scoring them on ENACT. Two facilitators assessed sessions independently on ENACT and compared their findings. Overall 90.9% level of satisfactory competence was achieved by the facilitators. Analysis of different domains of the ENACT showed that the facilitator developed good relationship with the women using effective non-verbal communication. However, there were certain domains in which facilitators could have done better such as asking the participants’ perspective of their problems, stimulating discussion, self-disclosure, using non-jargon terms and collaborative goal setting.

The majority of women reported that they were satisfied with the content, delivery format duration, and frequency of the sessions (Table 5, dimension IV). During online sessions, some participants, despite being encouraged to turn cameras on preferred keeping their camera off. This was identified as a potential barrier for implementation (IV.d.). Other identified barriers were lack of face-to-face interactions, occasional technical problems related to internet connection and lack of partner involvement in the sessions (IV.e, IV.g). To help participants overcome technical issues, support was offered by providing simple written instructions followed by a test session. This was reported to be helpful to overcome web-based challenges. The majority of the women suggested the content to have more information about baby care and birth (IV.a.) and some women suggested more flexibility with the time of the sessions’ delivery to overcome scheduling conflicts.

## Discussion

Considering successful achievement of recruitment and randomization, low levels of attrition at follow-up, and successful on-line delivery of THP in group setting, our findings indicate this preventive group intervention was feasible to be integrated into routine antenatal educational classes and it was acceptable to the participating women. At a personal level, it helped increase their psychosocial well-being, and self-confidence, while at the family and community levels, it helped address maternal-infant and marital relationships and improved social support. Similar findings have been reported from South Asia, where THP was delivered by peers (local lay women) through individual and groups sessions [48, 50] and was perceived acceptable and useful by most mothers. The study confirms the cross-cultural adaptability of the Thinking Healthy Programme and suitability for universal on-line group delivery.  

The quantitative findings showed small trends towards reduction in anxiety and depressive symptoms favoring the intervention arm, despite our study not being powered to detect differences between intervention and control conditions. Our pilot further showed that it was feasible to recruit and randomize women to intervention and control arms despite COVID-19 related administrative challenges and related delays, attrition rates were low at follow-up and the online delivery of THP-BGV in group setting through pregnancy school nurses was successful.

Our results add to the recent global review of evidence, conducted by the World Health Organisation (WHO), indicating moderate to strong effect sizes in favour of preventive interventions in reducing the severity of depressive and anxiety symptoms in the postnatal period [26, 51]. These reviews indicated that effects were strongest in interventions delivered to high-risk groups in the antenatal period, with cognitive behaviour therapy the most commonly employed approach. Based on this evidence, the Guidelines recommend preventive interventions in the antenatal period as part of maternal health care to prevent postnatal depression [15]. However, the WHO points out that the evidence for preventive interventions is mostly from High Income Countries, and recommends further research in LMICs.

To fill this gap, we believe the findings from our pilot study are sufficient to warrant a fully powered, large scale trial to evaluate the effectiveness of the adapted THP-BGV. The THP-BGV targeted all expectant women. For added feasibility and cost-effectiveness, future studies might, in line with WHO recommendations, focus the intervention to women at high risk for depression, e.g., women who have anxiety symptoms or sub-clinical depression so that resources are targeted to those who are most in need.

We found relatively low prevalence of perinatal depression in our sample (3.4%) compared to the rate of 19% indicated in a recent study in Turkey [4]. This could indicate that women with depression might not be accessing routine antenatal care and/or attending the online pregnancy schools [52, 53]. To increase the reach of the intervention, it would be important to raise community mental health awareness such as through media campaigns or community champions, and screening for all women who attend antenatal care.

The assessment tools used was well received and found helpful in raising women’s awareness about mental health and healthy life styles, as some participants stated during interviews. Epidemiological surveys from 15 countries showed mental disorders are as disabling as physical disorders. Higher disability of mental disorders compared to physical disorders was limited to disability in social and personal role functioning, rather than life activities [54]. Somatic symptoms might result from physiological changes in pregnancy, so the results of tools that measure disability such as WHODAS should be interpreted with caution.

The THP-BGV was delivered by nurses trained in the intervention. The participants found them acceptable and formed a good therapeutic relationship. Our findings add to the existing evidence that using a task shifting approach is a feasible and acceptable approach to increasing access to mental health interventions, and may be less stigmatizing than mental health professionals [55, 56]. However, training and supervision processes need to be strengthened, especially when scale-up of the intervention takes place.

The group format of the intervention provided participants an opportunity to validate, share and learn from each other’s experience. In a systematic review and thematic synthesis of the qualitative literature, women from the UK, Canada, Japan, and Australia, reported being validated of their experiences in a confidential and nonjudgmental environment helpful [11]. Normalization of PND by encouraging women to share their distress has also been highlighted in previous qualitative studies [57].

Our pilot study also identified barriers to participation in the study such as time constraints or lack of interest for the participants. Similar barriers have been reported in previous studies [51, 52]. In our study, the antenatal nurses gave only brief information about the study to the pregnant woman enrolled into the routine pregnancy schools. Providing more detailed information and the importance of psychosocial interventions could help improve participation. Likewise, offering incentives such as support with childcare, transport and refreshments can help to increase response rates, especially in face-to-face delivery.

The THP-BGV was delivered online, despite its convenience, accessibility, and flexibility, it might decrease the intervention effectiveness due to increased distractions, decreased participation, decreased facilitator control of the group, and difficulty to get feedback from the group required to tailor their sessions [58]. Even though the participants were satisfied with the online content, face to face classes could overcome the challenges mentioned above by decreasing distractions, encouraging more interaction among the group members, and increasing facilitator control of the group.

### Limitations of the study

It is notable that the reporting of some evaluation criteria, particularly those relating to the reasons behind unattendance was limited. Although THP had high acceptability, depression rates in the study sample was small, potentially suggesting limited interest or accessibility of the intervention for women with depression. Although all the women were from lower socioeconomic districts, all had access to technology. Online delivery will not address the digital divide in poorer women. Face to face delivery formats integrated into routine classes should also be tested.

The study does not provide information on challenges to scale-up, which are likely to be formidable. Future studies should also include information on cost-effectiveness to assist policy-makers in making an economic case for the intervention to be integrated into existing health systems.

## Conclusions

We conclude that this preventive intervention based on the well-established Thinking Healthy Programme is feasible and acceptable to stakeholders and warrants a definitive randomized trial to evaluate its effectiveness and cost-effectiveness in different settings.

## Supplementary Information

**Additional file 1.** Description of the assessment instruments.

## Data Availability

The datasets used and/or analysed during the current study are available from the corresponding author on reasonable request.

## Abbreviations

THP: Thinking Healthy Programme
THP-BGV: Thinking Healthy Programme – Brief Group version
EPDS: Edinburgh Postnatal Depression Scale
LMICs: Low and middle-income countries
PND: Perinatal depression
CMHCs: Community Mental Health Centers
HLCs: Healthy Lifestyle Centers
CBT: Cognitive Behavioral Therapy
mhGAP: Mental Health Gap Action Programme
MoH: Ministry of Health
MRC: Medical Research Council
RCT: Randomized controlled trial
RA: Research assistant
PI: Principal Investigator
ENACT: Enhancing Assessment of Common Therapeutic factors
PHQ-9: Patient Health Questionnaire-9
GAD-7: General Anxiety Disorder-7
COPE: Brief-Coping Orientation to Problems Experienced
MSPSS: Multidimensional Scale of Perceived Social Support
RAS: Relationship Assessment Scale
PSQI: Pittsburgh Sleep Quality Index
WHODAS: World Health Organization Disability Assessment Schedule
ORs: Odds ratios
CIs: 95% confidence intervals
