# Supplementary material for: Delivering the Thinking Healthy Programme as a universal group intervention integrated into routine antenatal care: a randomized-controlled pilot study
Source: BMC Psychiatry. 2023 Jan 6;23:14. doi: 10.1186/s12888-022-04499-6 (PMC9816542; doi:10.1186/s12888-022-04499-6)
Supplement: Supplementary file 1 — Additional file 1. Description of the assessment instruments. [file 12888_2022_4499_MOESM1_ESM.docx]

Methods

Assessment instruments

EPDS is a 10-item self-administered scale, developed for detection of symptoms of psychosocial distress during pregnancy and in the postnatal period. A score of >12 was considered as the cut-off [1,2,3].

PHQ-9 is a nine-item instrument which can diagnose depression as well as measure severity. A score of 10 or above indicates major depression, and scores of 5,10,15 and 20 represents mild, moderate, moderately severe, and severe depression respectively [4,5].

GAD-7 is a 7-item instrument that measures generalised anxiety disorder using a cut-off score of 10. The cut-off score was found to be 8 in the Turkish validation study [6,7].

The Brief COPE is a 28-item measure and assesses potentially important coping responses. The scales of the Brief-COPE included active coping, planning, positive reframing, acceptance, humor, religion, using emotional support, using instrumental support, self-distraction (mental disengagement), denial, venting, substance use, behavioral disengagement, self-blame. In the Turkish study suppression of competing activities and restraint coping were included, instead of active coping and self-blame [8,9].

The MSPSS is a 12–item self-report scale that was developed to measure perceived social support from family, friends, and significant other [10,11].

RAS is a brief 7 item scale, used to assess subjective satisfaction with a given relationship [12,13,14].

PSQI was developed to evaluate sleep quality in clinical studies. PSQI contains 19 self-rated questions and 5 questions rated by the bed partner if available. Self-rated items are combined to form seven component scores including; subjective sleep quality, sleep latency, sleep duration, habitual sleep efficiency, sleep disturbances, use of sleeping medication, and daytime dysfunction). The seven component scores are then added to yield one global score If the PSQI global score is greater than 5, it indicates poor sleep quality [15-16].

WHODAS 2.0 proxy administered form consisting of 12 items was used to measure disability and functional impairment. The questionnaire covers six domains of functioning: cognition (understanding and communication), mobility, self-care, getting along with others, life activities (work and household roles), and participation in society for the last month [17,18,19].

Higher scores on the Brief-COPE, MSPSS, RAS, and WHODAS indicate increased utilization of the specific coping strategy, higher levels of perceived social support, higher levels of relationship satisfaction, and higher disability of loss of function, respectively.

References:

1. Cox JL, Holden JM, Sagovsky R. Detection of postnatal depression: development of the 10-item Edinburgh Postnatal Depression Scale. Br J Psychiatry. 1987;150(6):782-786.
2. Engindeniz AN, Kuey L, Kultur S. Turkish version of the Edinburgh Postpartum Depression Scale. Reliability and validity study. Spring Symposiums I book. Psychiatric Organization of Turkey, Ankara.
3. Engindeniz AN, Kuey L, Kultur S. Validity and reliability of Turkish version of Edinburgh Postnatal Depression Scale. Book of Annual Meeting of Psychiatric Association of Turkey. Turkish Psychiatric Association Press, Ankara, 1996;51-2.
4. Kroenke K, Spitzer RL, Williams JB. The PHQ-9: validity of a brief depression severity measure. J Gen Intern Med 2001;16(9):606-613.
5. Sari YE, Kokoglu B, Balcioglu H, Bilge U, Colak E, Unluoglu I. Turkish reliability of the Patient Health Questionnaire-9. Biomedical Res India. 2016;27.
6. Spitzer RL, Kroenke K, Williams JBW, Löwe B. A brief measure for assessing generalized anxiety disorder: the GAD-7. Arch Intern Med. 2006,166(10):1092-1097.
7. Konkan R, Senormancı O, Guclu O, Aydin E, Sungur M. Validity and Reliability Study for the Turkish Adaptation of the Generalized Anxiety Disorder-7 (GAD-7) Scale. Archives of Neuropsychiatry. 2013,50(1):53-58.
8. Carver CS. You want to measure coping but your protocol’too long: Consider the brief cope. Int J Behav Med. 1997,4(1):92-100.
9. Bacanli H, Sürücü M, Ilhan T. An Investigation of Psychometric Properties of Coping Styles Scale Brief Form: A Study of Validity and Reliability. Educational Sciences: Theory Practice. 2013;13(1): 90-96.
10. Zimet GD, Dahlem NW, Zimet SG, Farley GK. The Multidimensional Scale of Perceived Social Support. J Personality Assesment. 1988;52: 30-41.
11. Eker D, Arkar H. Factorial structure, validity, and reliability of revised form of the multidimensional scale of perceived social support. Turkish J Psychology. 1995;34:45-55.
12. Hendrick SS. A generic measure of relationship satisfaction. Journal of Marriage and the Family. 1988,50(1):93-98.
13. Renshaw KD, McKnight P, Caska CM, Blais RK. The utility of the Relationship Assessment Scale in multiple types of relationships. Journal of Social and Personal Relationships. 2011,28(4):435-447.
14. Çelik E. Adaptation of Relationship Assessment Scale to Turkish Culture: Study of Validity and Reliability. International Journal of Psychology and Educational Studies. 2014,1:1-7.
15. Buysse DJ, Reynolds Iii CF, Monk TH, Berman SR, Kupfer DJ. The Pittsburgh Sleep Quality Index: a new instrument for psychiatric practice and research. Psychiatry Res. 1989,28(2):193-213.
16. Ağargün MY, Kara H, Anlar Ö. The validity and reliability of the Pittsburgh Sleep Quality Index. Turk J Psychiatry. 1996,7(2):107-115.
17. Ustün TB, Chatterji S, Kostanjsek N, Rehm J, Kennedy C, Epping-Jordan J, et al. Developing the World Health Organization Disability Assesment Schedule 2.0. Bull World Health Organ. 2010;88(11):815-823.
18. Aslan Kunt D, Dereboy F. Validity and Reliability of the World Health Organization Disability Assessment Schedule 2.0 (WHODAS 2.0) in Turkish Psychiatry Patients and Healthy ControlsSS. Turk Psikiyatri Derg. 2018,29(4):248-257.
19. Andrews G, Kemp A, Sunderland M, von Korff M, Ustun BT. Normative data for the 12 item WHO Disability Assessment Schedule 2.0. Plos One. 2009;4(12):e8343.
